# Collateral fattening in body composition autoregulation: its determinants and significance for obesity predisposition

**DOI:** 10.1038/s41430-018-0138-6

**Published:** 2018-03-20

**Authors:** Abdul G. Dulloo, Jennifer L. Miles-Chan, Yves Schutz

**Affiliations:** Department of Endocrinology, Metabolism and Cardiovascular System, Faculty of Science and Medicine, University of Fribourg, Fribourg, Switzerland

## Abstract

Collateral fattening refers to the process whereby excess fat is deposited as a result of the body’s attempt to counter a deficit in lean mass through overeating. Its demonstration and significance to weight regulation and obesity can be traced to work on energy budget strategies in growing mammals and birds, and to men recovering from experimental starvation. The cardinal features of collateral fattening rests upon (i) the existence of a feedback system between lean tissue and appetite control, with lean tissue deficit driving hyperphagia, and (ii) upon the occurrence of a temporal desynchronization in the recovery of body composition, with complete recovery of fat mass preceeding that of lean mass. Under these conditions, persistent hyperphagia driven by the need to complete the recovery of lean tissue will result in the excess fat deposition (hence collateral fattening) and fat overshooting. After reviewing the main lines of evidence for the phenomenon of collateral fattening in body composition autoregulation, this article discusses the causes and determinants of the desynchronization in fat and lean tissue recovery leading to collateral fattening and fat overshooting, and points to their significance in the mechanisms by which dieting, developmental programming and sedentariness predispose to obesity.

## From a historical perspective

There is a large body of work conducted over the last century on the energy budget strategies for growth in farm animals and laboratory rodents. In reviewing this field that encompasses nutrient partitioning, tissue growth and appetite control in mammals and birds, Webster [1] emphasized that food intake and the regulation of nutrient supply is to a large extent driven by the impetus for lean tissue growth. This is particularly striking in the congetically obese Zucker rats in which overeating is essential for achieving a normal rate of lean tissue growth, albeit at the expense of getting very fat in the process [2]. He went on to conclude that, to quote: ‘it may be both fair and kind to recognize the possibility that children with a predisposition to deposit excess fat may have a genuine extra hunger to achieve their target for lean tissue growth’ [1]. Although this contention has particular relevance for the mechanisms by which catch-up growth after faltered fetal/neonatal growth predispose to obesity [3–5], the essence of Webster’s conclusion can in fact be ascribed to what has more recently been referred to as ‘collateral fattening’—a process whereby excess fat is deposited as a result of the body’s attempt to counter a deficit in lean mass through overeating [6, 7]. Unlike Webster’s proposal, however, this more recent concept is not limited to the growth phase of the life cycle.

**Fig. 1:**
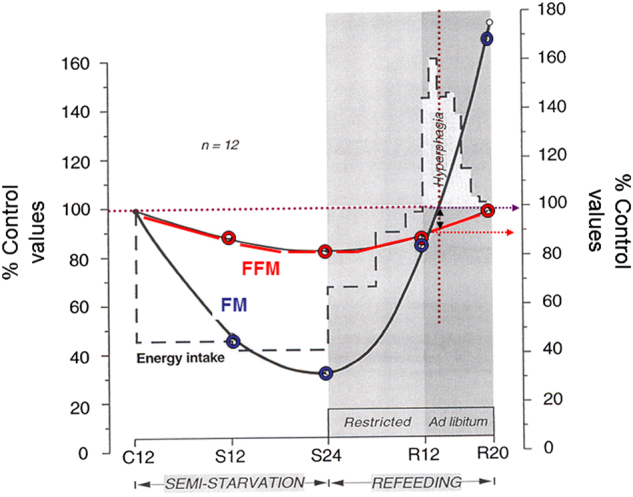
Dynamics of body composition changes in men participating in all phases of the Minnesota Experiment. The data are plotted to show the pattern of changes in energy intake, body fat mass (FM) and fat-free mass (FFM) during semistarvation and refeeding in the 12 men who completed all phases of the Minnesota Experiment (including the ad libitum phase of refeeding). All values are expressed as percentages of the control (pre-starvation) values. C12: end of 12 weeks of control period; S12 and S24: end of 12 weeks and 24 weeks of semistarvation respectively; R12 and R20: end of 12 weeks of restricted refeeding and 8 weeks of ad libitum refeeding, respectively. The double-headed arrow indicates that at the time-point when body fat had been fully recovered (i.e., 100% of control period value), FFM recovery is still far from complete, with hyperphagia persisting until completion of FFM recovery. Adapted from Dulloo et al. [9]

Indeed, the term collateral fattening derived from the reanalysis of the data on body composition and food intake from the Minnesota Starvation Experiment conducted in young men. In this classic longitudinal study lasting over a year at the end of World War II [8], the volunteer men of normal body weight were followed over a 12-week control (baseline) period before enduring 24 weeks of semistarvation, which resulted in a loss of ~25% of body weight, 70% of body fat and 27% of fat-free mass (FFM), the latter corrected for excess hydration. They were then refed in two distinct phases: a restricted refeeding phase lasting for 12 weeks, followed by another 8 weeks of refeeding on an ad libitum basis during which they developed marked hyperphagia lasting for several weeks. By the end of week 20 of refeeding, more body weight and fat were regained than were lost (Fig. 1)—a phenomenon that Keys and colleagues called ‘post-starvation obesity’ [8] and which nowadays is more commonly referred to as ‘fat overshoot’.

**Fig. 2:**
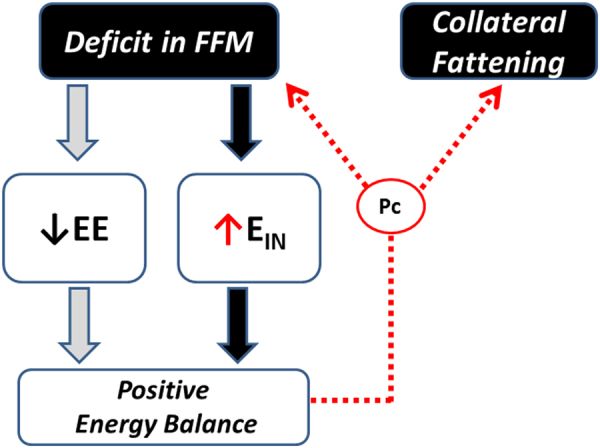
Concept of ‘collateral fattening’. A deficit in FFM results not only in a lower energy expenditure (EE) and hence lower energy needs for weight maintenance, but also in the activation a feedback loop that drives energy intake (E_IN_) in an attempt to restore FFM through the lean-to-fat partitioning characteristic (Pc) of the individual; reproduced from Dulloo et al. [7]

What these data on dynamic changes in body composition in relation to food intake revealed is that despite the complete recovery of body weight and fat mass, hyperphagia still persisted for some time until the complete restoration of FFM to the pre-starvation control (baseline) level (Fig. 1). Furthermore, the application of statistical analysis indicated that the degree of hyperphagia that occurred during the ad libitum refeeding phase is predicted by the degree of the FFM deficit independently of the fat mass deficit prior to ad libitum refeeding [9]. Taken together, these observations and analyses suggest that the compensatory hyperphagia that occurs in response to the loss of body weight is driven not only by the deficit in fat mass but also by the deficit in lean tissue. Thus, besides Kennedy’s lipostatic (or adipostatic) theory of food intake control whereby hyperphagia is a response to compensate for a deficit in fat mass [10], the possibility arises for the existence of a control system, which responds specifically to a deficit in lean mass through compensatory increases in energy intake. However, as lean tissue can only be gained with concomitant deposition of body fat (through the process of lean-fat partitioning), the process of completing the recovery of lean mass by overeating (after fat mass is fully recovered) is accompanied by an excess accumulation of body fat—hence collateral fattening leading to fat overshoot (Fig. 2). This process of collateral fattening is therefore a prerequisite for complete recovery of lean mass, and can thus be considered as a component of body composition autoregulation operating during weight recovery.

## Autoregulation of body composition during weight recovery

It is also evident that a prerequisite for collateral fattening (and fat overshooting) to occur is that there is a temporal desynchronization in the restoration of the body’s fat mass vs. lean mass, such that the recovery of fat mass reaches completion before that of lean mass. What then could be the causes or determinants of such desynchronization in fat and lean mass recovery that leads to collateral fattening?

In addressing this issue, it is important to emphasize that the synchronization of the recoveries of fat mass and lean mass in a way that these two masses reach complete (100%) recovery simultaneously would imply that the lean-fat partitioning characteristic of the individual during weight recovery is the same as during weight loss. In other words, the proportion of weight loss as fat (or as FFM) is equal to the proportion of weight recovered as fat (or as FFM), albeit after correction for any change in FFM hydration. Such temporal synchronization in fat and lean tissue recoveries underlies the existence of an intrinsic control of lean-fat partitioning—a contention first put forward by Payne and Dugdale [11]. It is supported by the findings from data on changes in body composition in the Minnesota Experiment indicating that despite the large inter-individual variability in the proportion of weight loss as lean tissue, the lean-fat partitioning characteristic of a given individual during the weight loss phase is conserved during the weight recovery phase [12]; the large inter-individual variability in lean-fat partitioning (expressed as the P-ratio—the fraction of body’s energy mobilized or deposited as protein during weight loss or recovery, respectively) being primarily determined by the pre-starvation (initial) % body fat [12, 13].

**Fig. 3:**
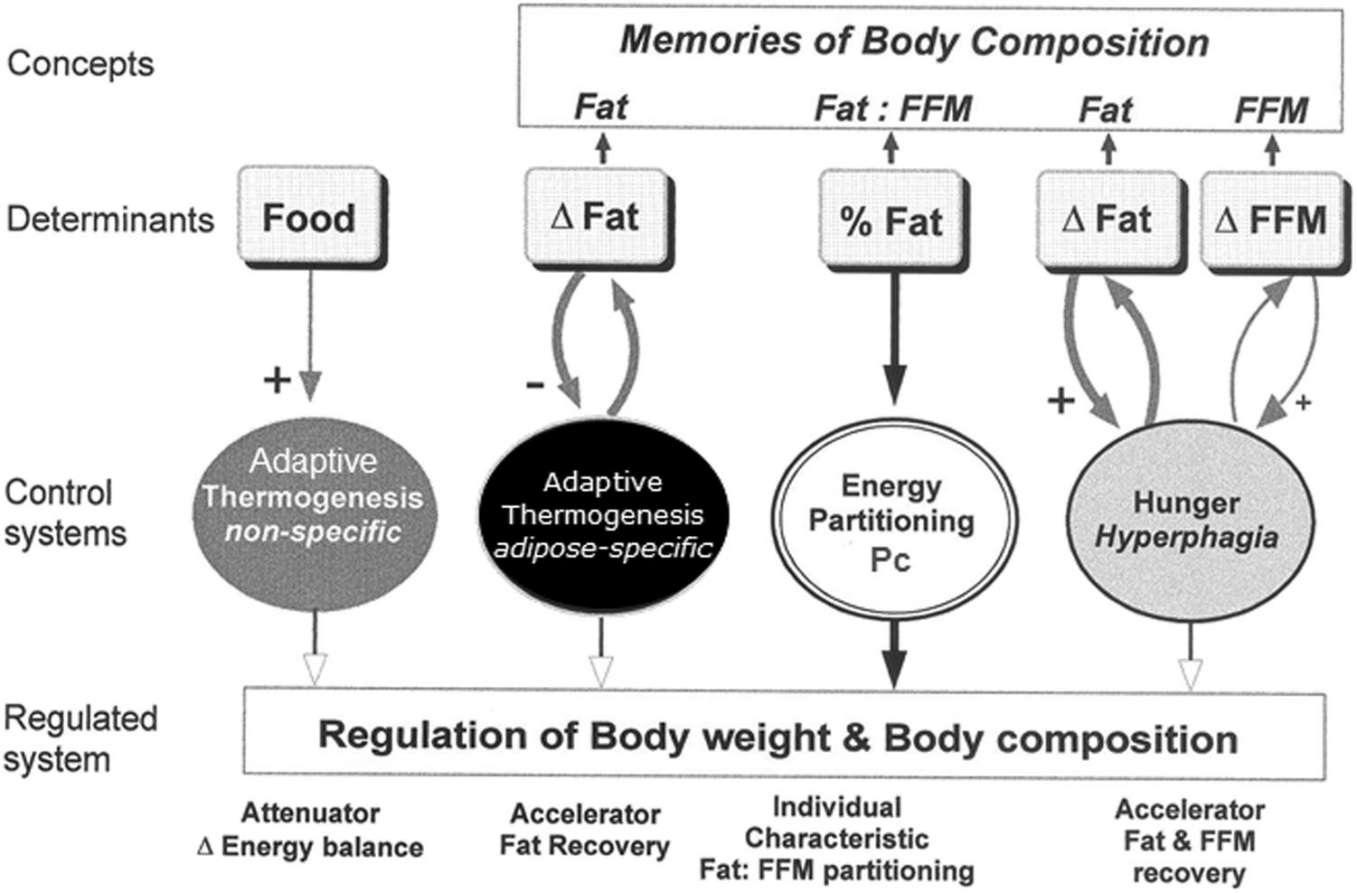
Conceptual model for autoregulation of body composition during weight recovery depicting the various control systems involved, namely: (i) the intrinsic control of energy partitioning between fat-free mass (FFM) and fat compartments, which determines the partitioning characteristic (Pc) of the individual as a function of initial percentage body fat (or fat: FFM ratio); (ii) the adipose-specific control of thermogenesis, which specifically accelerates fat recovery; (iii) the ‘non-specific’ control of thermogenesis, which functions as an attenuator of energy imbalance and is dictated by the food energy flux rather than by fat depletion, and (iv) the hunger-appetite drive leads to hyperphagia, the magnitude of which is determined by the extent to which body fat and FFM are depleted. Adapted from Dulloo and Jacquet [13]

This intrinsic control of lean-fat partitioning has been incorporated in a conceptual model of body composition autoregulation during weight recovery (shown in Fig. 3). It is constructed on the basis of results obtained from classic studies of starvation and recovery from starvation—in particular from the reanalysis of the several aspects of the Minnesota Experiment data on changes in body composition, food intake and basal metabolic rate [9, 12–14]. Its main features and operating modes are outlined below.

(I) The control of energy partitioning between lean and fat compartments confers a ‘basal’ or intrinsic energy partitioning characteristic (Pc) of the individual, and the initial body composition—i.e., the initial fat:FFM ratio—provides the individual with a ‘memory of partitioning’, which dictates the way fat and FFM are mobilized during weight loss and deposited during subsequent weight recovery [12, 13].

(II) The hunger-appetite drive leads to hyperphagia, whose magnitude is determined by the degree of depletion of both the fat and FFM compartments [9]. This hyperphagic response is thus dictated by a ‘memory’ of the initial fat mass, as well as FFM compartments. Within limits, the functional role of the hyperphagia is to accelerate the recovery of both fat and FFM according to the intrinsic partitioning characteristic of the individual.

(III) The adaptive suppression of thermogenesis that occurs during weight loss persists, at least in part, during weight recovery; its magnitude has been shown to be a specific function of the degree of fat mass depletion, but not that of FFM depletion [14]. This underscores the concept of a ‘fat-stores memory’ dictating an adipose-specific control of thermogenesis whose functional role is to accelerate specifically the body’s fat reserves and not FFM, thereby contributing to a disproportionate rate of fat recovery relative to that of lean tissue. It is distinct from the more rapid reacting ‘non-specific’ control of thermogenesis, which, under the control of the leptin/insulin–sympathetic–thyroid neurohormonal axis, functions as an attenuator of energy imbalance dictated by alterations in the food energy flux rather than by fat depletion per se [15].

## Causes and significance of temporal desynchronization in fat vs. FFM recovery

### Adaptive thermogenesis driving catch-up fat

**Fig. 4:**
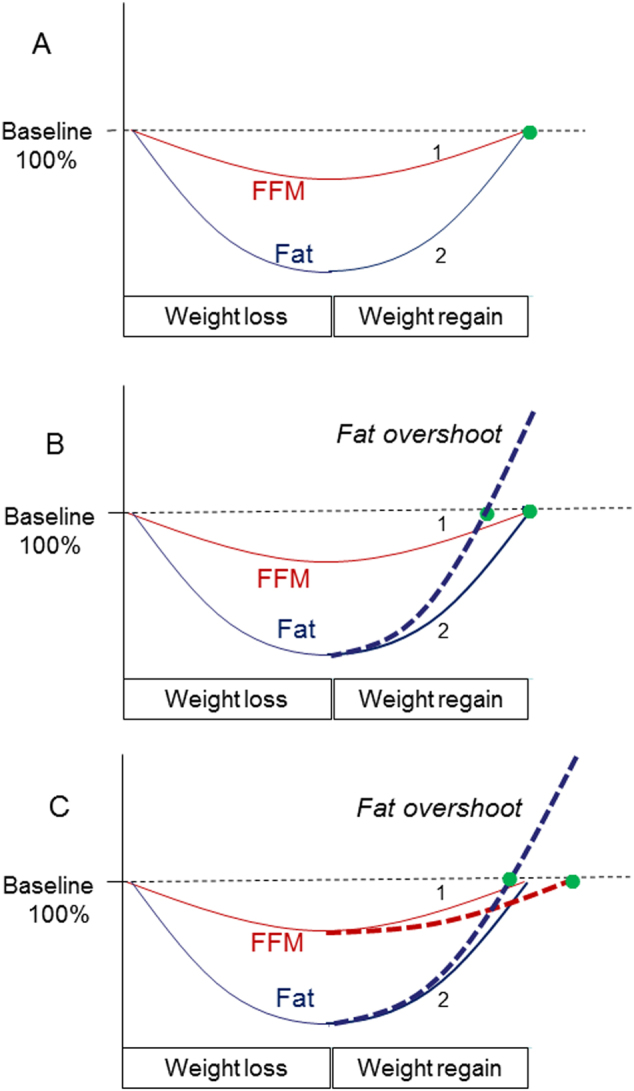
Schematic diagrams depicting dynamics of body composition recovery, with fat and FFM synchronization **a** or desynchronization **b**, **c** during weight recovery. The *Y* axis represent changes in fat and FFM as a percentage of initial (baseline) values, and the numbers 1 and 2 represent synchronized fat and FFM recoveries, respectively, as determined by the intrinsic lean-fat partitioning characteristic of the individual. **a** Fat and FFM reached complete (100%) recovery simultaneously; there is no fat overshoot. **b** Fat recovery alone is accelerated (either by adaptive thermogenesis or by excessive hyperphagia or by both) such that this catch-up fat results in complete (100%) fat recovery before complete FFM recovery; this desynchronization (represented by the gap between the green-filled circles) results in collateral fattening and fat overshoot. **c** Altered intrinsic lean-fat partitioning at the expense of FFM (i.e., slower recovery of FFM and hence energy diverted to recovery of fat) also results in complete fat recovery being reached before complete FFM recovery; this desynchronization (represented by the gap between the green-filled circles) results in collateral fattening and fat overshoot

A disproportionately greater recovery of fat relative to lean tissue (i.e., preferential catch-up fat) could, at least in part, be consequential of energy-conservation (thrifty) mechanisms operating specifically for accelerating fat recovery but not FFM recovery, leading to more fat being deposited in excess of that determined by the lean-fat partitioning characteristic of the individual. In this case, fat recovery would reach completion before that of FFM (Fig. 4, panel b vs. a).

Such a control system underlying preferential catch-up fat driven by suppressed thermogenesis is well described in animal models of controlled refeeding after caloric restriction [16, 17]. In humans, its existence is suggested from the reanalysis of data on dynamic changes in basal metabolic rate and body composition in the Minnesota Experiment. This showed that the reduction in mass-adjusted basal metabolic rate in response to weight loss was still present at the end of the 12-week phase of restricted refeeding, with the extent of this adaptive reduction in thermogenesis being specifically a function of fat mass depletion, but not that of FFM depletion [14]. A similar relationship between the magnitude of suppressed thermogenesis in the resting metabolic rate compartment and the recovery of fat mass (and not FFM) has been demonstrated in patients during nutritional rehabilitation from non-neoplastic gastrointestinal disease [18]. Furthermore, evidence for an adaptive suppression of thermogenesis in both the resting and non-resting compartment of daily energy expenditure driving catch-up fat can also be drawn from data on humans undergoing weight recovery after 2 years of sustained caloric restriction (resulting in 15% weight loss) in the Biosphere 2 Experiment [19]. Taken together, the common observation in all these studies showing preferential catch-up fat in part through suppressed thermogenesis is that body fat recovery reaches completion well before FFM is fully recovered.

It is to be noted that this adipose-specific control of thermogenesis underlying preferential catch-up fat has been observed only after substantial depletion of fat mass—as in the case of the studies mentioned above—where fat depletion represented more than a third of pre-starvation fat levels. Indeed, in a recent study by Müller et al. [20], whereby the degree of fat depletion induced by caloric restriction for 3 weeks was mild (~6% relative to baseline ‘habitual’ levels), the phenomenon of preferential catch-up fat was not observed. Taken together, it would seem that fat depletion would need to exceed a ‘threshold’ level for the occurrence of adaptive thermogenesis driving preferential catch-up fat and eventually resulting in fat overshooting. This adipose-specific control of thermogenesis driving catch-up fat therefore constitutes an adaptive mechanism that accelerates the restoration of survival capacity (conferred by the body’s fat reserves) toward withstanding the next period of food scarcity. However, it also contributes to a desynchronization in fat and FFM recoveries. Besides adaptive thermogenesis, there are other potential explanations as to why temporal desynchronization in the recoveries of the fat and FFM occur; these are discussed below.

### Excessive hyperphagia

**Fig. 5:**
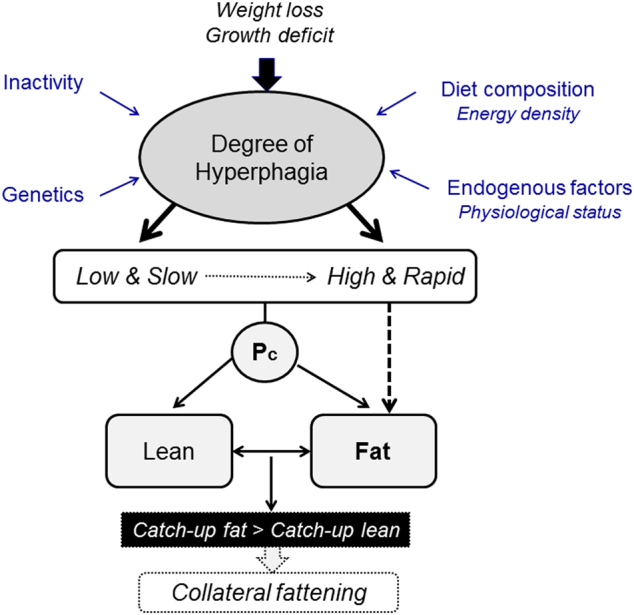
Conceptual overview of the factors contributing to hyperphagia, which could operate at different rates as well as with different magnitude. Lean (protein) tissue synthesis and retention may lag behind fat tissue synthesis and storage, because of the more complex and differently regulated metabolism in the former, as well as its limited deposition, in particular in non-stimulated muscles, that is, without associated exercise. Despite a constant intrinsic partitioning characteristic (Pc), more body fat can be deposited in situation of high and rapid degree of hyperphagia leading to faster catch-up fat relative to lean tissue, temporal desynchronization in reaching complete fat vs. lean mass recovery, and collateral fattening resulting in fat overshoot and altered body composition with a higher degree of adiposity

The degree of hyperphagia may be too high so that the capacity for increasing the lean tissue synthesis rate is exceeded. Unlike the very high capacity for fat accumulation in the body, the rate of lean tissue deposition is limited, and under conditions of excessive caloric load—as may occur during refeeding on modern diets, that is, refined energy-densed foods low in bulk and hence with low satiation—more body fat is deposited than could be determined by the intrinsic partitioning characteristic of the individual (Fig. 5). In this case also, one can refer to a situation of a preferential catch-up fat, thereby resulting in temporal desynchronization in reaching complete fat and FFM recovery and consequential collateral fattening (Fig. 4, panel b).

### Impaired lean tissue growth

A desynchronization in fat and FFM recoveries can also occur as a result of specific impairments in the rate of lean tissue deposition, and exacerbated by the fact that the energy spared as a result of diminished lean tissue gain is diverted to more fat gain—that is, a state of ‘double burden’ on the synchronization of lean and fat tissue recovery (Fig. 4, panel c). Among several factors that could account for this are situations where refeeding diets do not meet the requirements for lean tissue growth such as inadequate quantity or quality of dietary protein, deficiencies in growth-promoting micronutrients such as zinc and vitamin A, or due to suboptimal stimulus for muscle growth resulting from insufficient muscle contractility, as may arise from a lack of physical activity.

### Altered control of partitioning: relevance to aging and developmental programming

One may also entertain the possibility of a change in the intrinsic lean-fat partitioning characteristic of the individual during the weight recovery phase such that a lower lean-fat partitioning will favor a greater fat regain at the expense of less lean tissue deposition. This could be postulated to occur with the aging process, which would be consistent with the report that in postmenopausal overweight/obese older women (age ~ 60 years) who were followed through a 5-month weight loss intervention and a subsequent 12-month non-intervention period, those who regained weight (≥2 kg) regained more than two-thirds of the fat lost but only 25% of lean mass lost [21].

Alterations in the intrinsic lean-fat partitioning characteristic could also result from epigenetic changes resulting from developmental (fetal or neonatal) programming. Indeed, a preferential catch-up fat with lean tissue lagging behind is also a risk factor for obesity and insulin-related complications in infants and children who experienced catch-up growth after perinatal growth retardation. In addition to some evidence for thrifty metabolism [4]—which seems to persist in adulthood [22]—there is also evidence based upon early feeding studies in infants [23] that post-natal catch-up growth may also be attributed to hyperphagia, which may be driven by the impetus to catch-up on the lean mass lagging behind fat mass recovery. This may thus lead to excess fat deposition through collateral fattening that may extend for years and contribute to their increased risk for obesity in later life.

## Importance of initial adiposity and relevance to dieting making one fatter

**Fig. 6:**
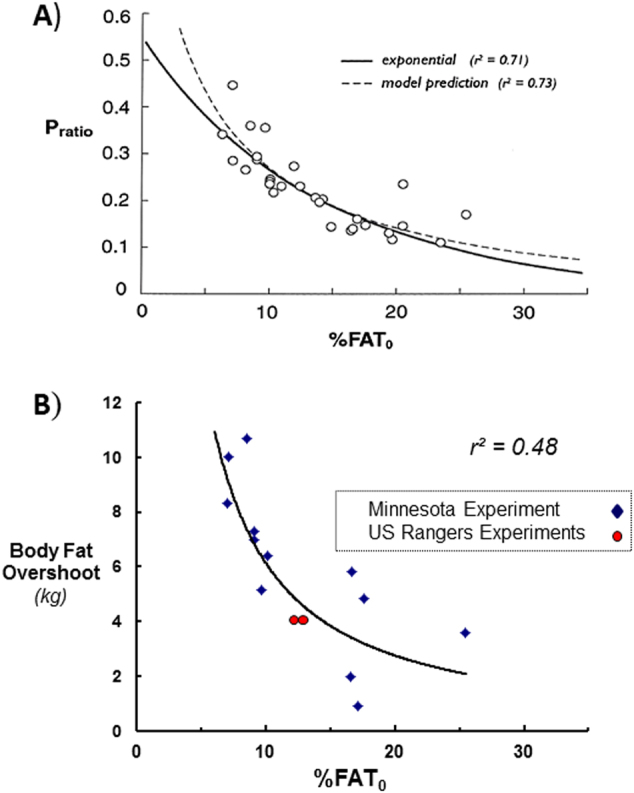
**a** Relationship between the proportions of energy mobilized as protein (P-ratio) during weight loss due to semistarvation and the initial percentage body fat (%FAT0) in men of the Minnesota Experiment. Note that P-ratio (expressed in energy terms) is a proxy of the fraction of weight loss as FFM (i.e., ΔFFM/ΔWeight). Adapted from Dulloo and Jacquet [13]. **b** Relationship between the extent of fat overshooting (kg excess fat regained) and the initial (pre-starvation) percentage body fat (%FAT0). The exponential curve is drawn from data on the 12 men who participated in all phases of the Minnesota Experiment (data for each individual is represented by a blue-filled diamond symbol). The mean values for men (*n* = 10) participating in each of the Army Ranger training experiments for which body composition data are available [25, 26] are shown as red-filled circles. Adapted from Dulloo et al. [24]

From a standpoint of body composition autoregulation in healthy young subjects, it would seem that the desynchronization between lean and fat tissue recoveries would, to a great extent, be determined by preferential catch-up fat (Fig. 4 panel b), the magnitude of which is determined by the degree of fat and FFM depletion with impact both on hyperphagia and suppressed thermogenesis. It can be argued that for the same loss in body weight (in absolute amounts or as a percentage of initial weight), a lean person compared with a fatter person would show greater degrees of fat and lean tissue depletions, as well as a greater proportion of weight loss as lean tissue (Fig. 6, panel a). Under these conditions, it therefore follows that the leaner the individual the greater the temporal desynchronization in fat and lean tissue recoveries, and hence the greater the fat overshoot [24]. Support for this contention can be derived from the data on body composition in the men of the Minnesota Experiment in which, by virtue of the study design, a similar degree of weight loss was achieved in all participants, namely a loss of 25–29% of initial body weight. As shown in Fig. 6 (panel b), a plot of the amount of fat overshot against pre-starvation (initial) %body fat reveals an exponential increase in the extent of fat overshoot with decreasing initial % body fat [24]. Also superimposed on this figure are the data from U.S. Army Rangers who, during rehabilitation after losing about 12% of their weight due to training in a multi-stressor environment (including energy deficit through food deprivation), showed substantial fat overshoots of 4–5 kg on average [25, 26]; data on their average amount of fat overshoot and average baseline body fat% fit closely with the exponential curve drawn from the Minnesota Experiment data.

Such an inverse relationship between fat overshoot and initial adiposity suggest that dieters with normal body weight are at higher risk for fat overshooting than overweight/obese dieters, and that repeated dieting and weight cycling put the lean person rather than the overweight/obese person at greater risks toward becoming fatter [24], as well as increased cardiometabolic risk [27]. An explanation based upon collateral fattening would be consistent with the analysis of several prospective studies [28, 29] indicating that it is dieting among those individuals with perceived overweight (but actually of normal weight), rather than in those who are actually overweight or obese, which most strongly and consistently predict future weight gain and obesity (reviewed in refs. Dulloo et al. 24 and Montani et al. 27).

## A link between sedentariness and obesity predisposition

Collateral fattening could also be invoked among the mechanisms by which a marked reduction in physical activity in previously active individuals may predispose them to increased adiposity, beyond that explained by diminished physical activity energy expenditure. It is now increasingly recognized that a J-shaped relationship exists between physical activity levels and energy intake [30, 31], with energy intake tightly coupled to energy expenditure across moderate to high activity levels thereby promoting energy balance, and dysregulation occurring at low levels of physical activity. Indeed, an increase in sedentariness may not lead to compensatory decrease in energy intake, thereby predisposing the individual to increased adiposity and weight gain. One reason for such intake–expenditure mismatching may be attributed to the fact that with increasing sedentariness, the resulting reduction in muscle contractile function can lead to subsequent loss of muscle tissue and hence a deficit in lean mass. Indeed, in healthy young men, short periods of muscle disuse arising from strict bed rest for <10 days have been shown to lead to a reduction in skeletal muscle mass and substantial loss of lean body mass [32]. Based upon the concept of collateral fattening, a potential feedback system attempting to restore such deficit in lean tissue may lead to a stimulatory effect on energy intake thereby contributing to the intake–expenditure mismatching, positive energy balance and concomitant increase in adiposity.

## Concluding remarks

Over the past few years, there has been a resurgence of interest into the role of body composition in the control of appetite/hunger, with particular focus on the role of FFM [33–39]. Various models are being proposed to integrate the functional relationships of both fat mass and FFM with the control of energy intake [40–42], with the model of Stubbs et al. [43] (in a companion paper in this issue) taking into account the ‘V’- or ‘U’-shaped relationship between appetite/hunger and FFM [7]. Indeed, in examining the role of FFM in the control of food intake, we have previously emphasized the distinction between a ‘passive’ vs. an ‘active’ role of FFM [7]: the passive role being mediated by ‘energy-sensing’ mechanisms that translate FFM-induced energy requirements to energy intake, and the active role operating in the defense of FFM deficit by driving hyperphagia. Thus, the loss of FFM or its deficit resulting from dieting, developmental programming or sedentariness should be regarded not only as contributing to increased adiposity because of the lowering of maintenance energy expenditure due to a lower FFM, but also to the body’s attempt to restore FFM by overeating. This concept of collateral fattening in body composition autoregulation provides a system physiology framework in the search for peripheral signals (proteinstats) linking lean tissue and food intake. It also serves to emphasize further the importance of a healthy lifestyle centered on balanced diets and physical activity in the protection against lean mass deficits pertaining to both the prevention and treatment of obesity.
